# Central Coherence in Eating Disorders: A Synthesis of Studies Using the Rey Osterrieth Complex Figure Test

**DOI:** 10.1371/journal.pone.0165467

**Published:** 2016-11-02

**Authors:** Katie Lang, Marion Roberts, Amy Harrison, Carolina Lopez, Elizabeth Goddard, Mizan Khondoker, Janet Treasure, Kate Tchanturia

**Affiliations:** 1 King’s College London (KCL), Psychological Medicine, Institute of Psychiatry, Psychology & Neuroscience, London, United Kingdom; 2 Regents School of Psychotherapy & Psychology, Faculty of Humanities, Arts & Social Sciences, Regent’s University, London, United Kingdom; 3 Department of Pediatrics and Child Surgery East, Faculty of Medicine, University of Chile, Santiago, Chile; 4 Illia State University, Department of Psychology, Tbilisi, Georgia; Universita degli Studi di Udine, ITALY

## Abstract

**Background:**

Large variability in tests and differences in scoring systems used to study central coherence in eating disorders may lead to different interpretations, inconsistent findings and between study discrepancies. This study aimed to address inconsistencies by collating data from several studies from the same research group that used the Rey Osterrieth Complex Figure Test (Rey Figure) in order to produce norms to provide benchmark data for future studies.

**Method:**

Data was collated from 984 participants in total. Anorexia Nervosa, Bulimia Nervosa, recovered Anorexia Nervosa, unaffected family members and healthy controls were compared using the Rey Figure.

**Results:**

Poor global processing was observed across all current eating disorder sub-groups and in unaffected relatives. There was no difference in performance between recovered AN and HC groups.

**Conclusions:**

This is the largest dataset reported in the literature and supports previous studies implicating poor global processing across eating disorders using the Rey Figure. It provides robust normative data useful for future studies.

## Introduction

Research conducted with individuals with Anorexia Nervosa (AN) has highlighted the presence of an inefficient cognitive processing style [1,2]. This is now thought to be a shared characteristic (to varying degrees) across eating disorder sub-types, and a likely contributor to their pathogenesis [3].

Central coherence is one such area of interest in the neuropsychological study of eating disorders (ED). Historically, central coherence is grounded in Gestalt psychology, which hypothesises that integrated coherent structures form the basis of processing and perception [4]. It also posits that the structured whole is different from the sum of the configural parts. Following gestalt theory, Navon’s global precedence hypothesis theorised that information processing follows a hierarchical network, from global structures to more local structures, whereby global elements take precedence. Importantly, this theory suggests that global and local processing are not independent, and interact to provide the whole unit [5].

Research has highlighted that individuals with eating disorders (ED) may not process information in the same hierarchical fashion described above. There is a wealth of research demonstrating that adults with AN have poor global processing (for a systematic reviews see (Lang et al., 2014; Lopez et al., 2008)), often with superior detail focused processing [6]. This profile of weak central coherence is also present once weight has been restored and in children and adolescents, albeit in an attenuated form [7–9]. Furthermore, this processing style seems to have a familial component, as unaffected relatives of those with AN, such as mothers and sisters, also demonstrate poor global processing [9–11].

Although there is less research, studies with individuals with Bulimia Nervosa (BN) have also demonstrated poor global processing in comparison to healthy controls (HCs); suggesting that this processing style is a trans-diagnostic characteristic amongst EDs [9,12,13].

Though poor global processing does not seem to be specific to the EDs and can be seen as a common mechanism across other psychiatric disorders such as Autism Spectrum Disorder [14], Schizophrenia [15] and Obsessive Compulsive Disorder [16], it does appear to be present most consistently in ED, and certainly the pattern of heritability of these traits seems stronger [17,18].

A current methodological problem in the field of neuropsychology of ED is the large variability in the number of tests used to measure central coherence together with differences in scoring methods, which may be contributing to conflicting results between studies. It is therefore of upmost importance that future studies employ robust neuropsychological tests and valid scoring methods to allow for accurate assessment of central coherence in ED.

The Rey Osterrieth Complex Figure Test (Rey Figure, [19]) is a popular neuropsychological measure of central coherence used in the ED and wider psychiatric field. It is a pencil and paper task whereby the participant is asked to make a direct copy of a complex figure. The way in which the participant draws the figure can offer insights into their processing style. Historically, participants were asked to make a direct copy of the shape and also one from memory and both the copy and the delayed recall were used to measure central coherence. However, the most direct measure of central coherence is now considered to be collected from the direct copy using Booth’s (2006) scoring system [20]. This scoring system incorporates both the order in which the participant chooses to draw the elements (whether preference is shown to global or detailed elements) and the style in which they are drawn in (fragmented or coherent). Full administration and scoring instructions can be found at www.katetchanturia.com (http://www.katetchanturia.com/#!clinical-work-packages—protocols/cff4).

Studies employing the Rey Figure have consistently demonstrated poor global processing in AN, BN, weight restored AN and also healthy unaffected relatives of those with AN compared to HCs [2,7–9,12]. With this in mind, the present study aims to collate data on the Rey Figure in ED from several studies containing participants with AN, BN, recovered AN, unaffected family members and healthy controls, using Booth’s (2006) scoring system. Collating data in this fashion rather than performing a meta-analysis will allow us to provide normative comparison data for future studies. A secondary aim of the study is to investigate possible predictors of central coherence across the different ED sub-types.

## Method

### Participants & procedure

Data were drawn from six previously published studies and two unpublished datasets carried out within the Eating Disorders Research Unit, King’s College London [6,7,9,21–23]. The collated dataset consisted of 984 female participants (age ranging between 18–65 years) with either AN (N = 364), BN (N = 125), or individuals who had recovered from AN (N = 107), unaffected relatives (N = 85 mother and N = 30 sisters) of individuals with AN, and HCs (N = 273). Data were collected between 2007 and 2014. In all studies, participants with an ED were recruited from the specialist ED service of the South London and Maudsley NHS Trust and the community. HCs were recruited via advertisements in the local community.

Semi-structured interviews were used across the different studies, such as the SCID [24] and the EATATE [25] phenotype interview to determine the different ED diagnoses of the participants.

Individuals with AN (either restricting subtype or binge/purge subtype) were included in the studies if they had a body mass index (BMI) of 17.5 or below. The BN group consisted of individuals with a binge/purge frequency of more than twice a week for the past three months in line with DSM-IV criteria. The recovered group consisted of female participants who had recovered from AN and reported restored, regular menstruation for at least the previous year, did not report clinically significant scores (4 or above) on the Eating Disorders Examination Questionnaire [26], and had a BMI of 18.5 or above for at least the previous year.

Unaffected relatives were N = 85 mothers of adolescent daughters with AN and N = 30 sisters of adult sisters with AN with no current or prior history of an ED or other psychiatric illness themselves. There were no significant differences on any of the indices of the Rey between unaffected mothers or sisters and therefore the two groups were collapsed into one ‘unaffected relatives’ group.

HCs had no current or prior history of psychiatric illness. They were excluded if they reported a family history of an ED. Exclusion criteria for all groups were a learning difficulty, neurological impairment, or psychosis. All studies had ethical approval from the NHS national committee (ethics numbers: 020/05; 12/LO/2015; CREC/07/08-67, 08/H0606/58; 08/H0306/66), and all participants had given written informed consent.

#### Measures

*Demographic information*: Each of the collated studies had used a questionnaire to collect demographic information. This included: age, medication status, duration of illness, number of years of education and ethnicity.

*The Structured Clinical Interview for DSM-IV* (SCID, [24]) screening questionnaire was implemented to screen for the presence of possible psychiatric illnesses within both the ED and HC groups. The SCID is a semi-structured interview that probes for symptoms of depression, anxiety, OCD, substance abuse and ED pathology. It is widely used and recommended for research protocols in psychiatric populations.

*EATATE phenotype interview* [25]: A semi-structured diagnostic interview that assesses life time course of psychiatric disorders.

*Self-reported anxiety and depression* were measured using either the Hospital Anxiety and Depression Scale (HADS [27], 89% of participants) or the Depression, Anxiety and Stress Scale (DASS [28] 11% of participants). As different measures of anxiety and depression were employed between the studies, outcome scores were standardised by calculating z scores for the depression and anxiety scale of each measure.

*Rey Osterrieth Complex Figure Test* [19]: The Rey Figure is a pencil and paper neuropsychological task commonly used in both the ED and wider psychiatric field as a measure of gestalt processing. The drawing strategy adopted by the participant is used as a measure of central coherence. Given the task instructions to copy the figure “as carefully as you can”, performance in terms of accuracy is benefitted from taking a more global approach. Participants are asked to start drawing using a black coloured pencil, and the experimenter changes the coloured pencil each time one of the 18 elements are completed or if the participant begins drawing a new element without completing the previous one. The specific order in which the pencils are used (black, green, purple, brown, dark blue, pink, light blue, red, yellow and orange), aids with scoring of the Rey Figure once the drawing has been completed. The administration of all participants Rey Figures were also video recorded. This further aids with the reliability of the scoring, and allows for each Rey Figure to be scored by a second and independent researcher.

**Fig 1:**
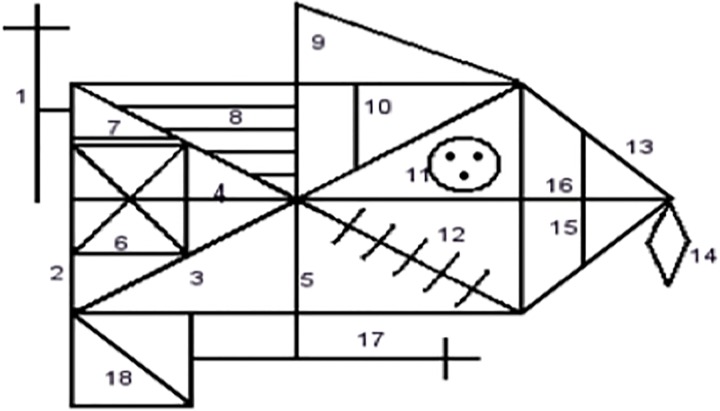
Scoring elements for the Rey Figure.

The Rey Figure was scored according to a slightly modified version of Booth’s (2006) scoring method (amendments to the scoring method were discussed with R. Booth prior to administration). The order index was modified so that the detail of the diagonal line of element 18 was not required. Two modifications to the style index were made. Firstly, whereas full extension of the vertical line (element 5) was previously required, this was modified so that only extending the line above or below would result in full marks. Secondly, the requirement to also draw element 15 with element 13 was also eliminated. This method incorporates both the order in which the participant chooses to draw the elements (whether preference is shown to global or detailed elements) and the style in which they are drawn in (fragmented or coherent). Order index and style index are computed and added to give the Central Coherence Index (CCI). Booth’s (2006) scoring method is hierarchical, whereby higher scores are awarded when preference is shown to global elements. Higher scores on this measure are indicative of better global or holistic processing. An outline of Booth’s scoring system is provided in Table 1 and Fig 1. A second researcher co-rated 10% of the Rey figure data for each dataset and the reliability of the datasets ranged from 0.71–0.97.

**Table 1:** Scoring system for Rey Figure according to Booth (2006).

Category	Element	Description
Global element (Score = 4)	2	Large rectangle
	13	Side of large triangle attached to large rectangle
Global internal element (Score = 3)	3	Diagonal cross
	4	Horizontal midline of large rectangle
	5	Vertical midline of large rectangle
	16	Horizontal line with sides of large triangle
Local perimeter element (Score = 1)	1	Vertical cross
	9	Small triangle above large rectangle
	14	Diamond
	17	Horizontal cross
	18	Square attached to large rectangle
Local internal element (Score = 0)	6	Small rectangle
	7	Small horizontal line above small rectangle
	15	Vertical line within side of large triangle
	8	Four parallel lines
	10	Small vertical line with large rectangle
	11	Circle with three dots
	12	Five parallel lines

#### Statistical analysis

Histograms were used to assess the data for normality. Both the order and the Central Coherence Indices of the Rey Figure were negatively skewed and so reverse log transformations (whereby scores are first reversed and then a log transformation is applied) were employed to normalise the data when testing for group differences.

Regression analysis was employed to investigate group differences in order index, style index and CCI. A secondary regression analysis was employed to assess potential predictors of CCI within each ED group. Due to the testing of multiple variables, we used a more conservative significance level of 1%, to minimise Type I errors. Cohen’s d was calculated to provide effect sizes (>0.5 = moderate; >0.8 = large). Data was analysed using the statistical package IBM SPSS version 22.00.

## Results

### Demographic data

Table 2 displays demographic information for each group. In general, clinical groups were in their mid-late 20s, and had a long duration of illness (more than 9 years). Those with current AN were significantly underweight (mean BMI 15.7) while all other groups had a BMI within the normal range. There was a significant different in age between the HC group and the AN recovered group and unaffected relative groups, therefore analyses were adjusted for age.

**Table 2:** Participant demographics.

	Age**	BMI**	Illness duration	Years of education**	Medication (% yes)	Anxiety*	Depression*
AN (N = 364)	26.1 (8.1) *p* = .290 d = 0.01	15.6 (2.1) *p* < .000 d = 2.98	9.0 (7.8)	15.4 (2.4) *p* < .001 d = 0.39	46%	0.6 (1.3) *p* < .001 d = 1.16	0.6 (0.4) *p* < .001 d = 3.0
BN (N = 125)	27.0 (7.7) *p* = .857 d = 0.01	21.6 (2.3) *p* = .780 d = 0.0	10.3 (7.6)	15.8 (2.5) *p* = .097 d = 0.22	20.7%	0.6 (0.7) *p* < .001 d = 2.11	0.3 (0.7) *p* < .001 d = 1.76
AN Recovered (N = 107)	29.4 (10.6) *p* = .026 d = 0.28	20.8 (2.0) *p* = .001 d = 0.42	9.4 (7.3)	16.2 (2.5) *P =* .792 d = 0.04	8.3%	-0.6 (0.5) *p* < .001 d = 0.0	0.0 (0.5) *p* < .001 d = 1.4
Unaffected Relative (N = 115)	44.5 (13.3) *p* < .000 d = 1.75	23.0 (3.2) *p* = .001d = 0.59	-	15.8 (3.0) *p* = .139d = 0.20	-	-0.2 (0.9) *p* < .001 d = 0.62	-0.3 (0.9) *p* = .001 d = 0.51
HC (N = 273)	26.9 (8.4)	21.6 (1.9)	-	16.3 (2.2)	-	-0.6 (0.5)	-0.6 (0.4)

AN = Anorexia Nerovsa; BN = Bulimia Nervosa; AN recovered = Recovered Anorexia Nervosa; HC = Healthy Control; BMI = Body Mass Index.

*Z score calculated from the Hospital Anxiety and Depression Scale or the Depression, Anxiety and Stress Scale.

**P value and effect size when compared to HC group.

### Rey Figure: Normative data

Medians and interquartile ranges for each outcome of the Rey Figure stratified by clinical group are displayed in Table 3.

**Table 3:** Medians and interquartile ranges for normative data for the Rey Figure.

	Normative data		
	Order Index median	Style Index median	Central Coherence Index median
AN (N = 364)	2.17 (1.50–2.50)	1.50 (0.93–1.67)	1.38 (1.02–1.61)
BN (N = 125)	2.17 (1.83–2.54)	1.33 (1.00–1.67)	1.36 (1.09–1.60)
AN Recovered (N = 107)	2.17 (1.78–2.67)	1.33 (0.90–1.67)	1.41 (1.05–1.66)
Unaffected Relative (N = 115)	2.17 (1.50–2.50)	1.33 (0.98–1.67)	1.34 (1.02–1.53)
HC (N = 273)	2.33 (1.97–2.67)	1.50 (1.16–1.67)	1.50 (1.21–1.68)

AN = Anorexia Nerovsa; BN = Bulimia Nervosa; AN recovered = Recovered Anorexia Nervosa; HC = Healthy Control.

### Rey Figure: Group comparisons

**Fig 2:**
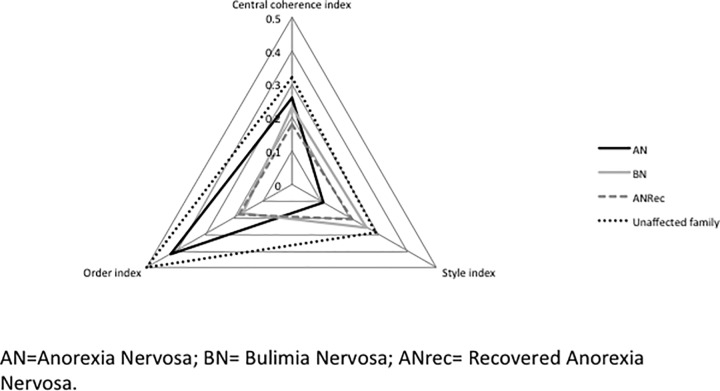
Radar chart of effects sizes of each of the indices of the Rey for each group in comparison with HC group.

Table 4 displays the means, transformed means (used for analysis where the data were skewed) and standard deviations and medians for the Rey Figure outcome variables. AN, BN and unaffected relative groups differed significantly from HC on all three Rey Figure outcome variables. No significant differences were found for the AN recovered group. Fig 2 depicts a radar chart of the effects sizes of each of the indices of the Rey for each group.

**Table 4:** Means and transformed means (where data were skewed) and Standard Deviations for the Rey Figure analysis.

	Order Index	Style Index	Style Index transformed*	Central Coherence Index	Central Coherence Index transformed*
AN (N = 364)	1.98 (0.72) *p* < .001 d = 0.42	1.32 (0.50)	0.47 (0.30) *p* < .004 d = 0.11	1.28 (0.43)	0.51 (0.24) *p* < .001 d = 0.26
BN (N = 125)	2.16 (0.58) *p* = .026 d = 0.17	1.30 (0.43)	0.50 (0.26) *p* = .003 d = 0.26	1.31 (0.34)	0.50 (0.20) *p* = .001 d = 0.23
AN Recovered (N = 107)	2.15 (0.66) *p* = .780 d = 0.18	1.30 (0.46)	0.49 (0.27) *p* = .090 d = 0.21	1.32 (0.40)	0.49 (0.23) *p* = .187 d = 0.18
Unaffected Relative (N = 115)	1.95 (0.64) *p* < .001 d = 0.50	1.27 (0.47)	0.51 (0.28) *p* < .001 d = 0.29	1.27 (0.39)	0.52 (0.22) *p* < .001 d = 0.32
HC (N = 273)	2.26 (0.61)	1.39 (0.43)	0.44 (0.22)	1.40 (0.36)	0.45 (0.22)

*Data have been transformed using reverse log transformations, therefore value of mean has been reversed.

P value and effect size when compared to HC group

Analysis performed using regression analysis.

### Predictors of central coherence within ED subtype

Regression analyses were conducted within each ED group to investigate possible predictors of central coherence (age, illness duration, BMI, years of education and anxiety and depression). Separate regressions were performed within each sub-group as data for some of the covariates were not available for the HC sample.

None of these variables significantly predicted CCI outcome in AN or AN recovered. The lack of statistical significance for these analyses may be due to the small sample within some of the sub-groups.

## Discussion

This study assessed central coherence in ED by collating data from previously conducted studies within the same research group that used the Rey Figure with psychologists trained to use a consistent administration and scoring system to measure central coherence [20], and applied it systematically and reliably to individuals with AN, BN, recovered AN, unaffected relatives of AN and HCs. Secondly, we aimed to provide benchmark data using this robust measure and consistent scoring method with a large number of participants across different ED diagnoses.

The results of this study were consistent with previous research, demonstrating poorer global processing in AN, BN and unaffected AN relatives in comparison to HCs as evidenced by lower scores on central coherence indices [6,10,22,29–31]. However, the findings are discrepant with a number of studies that report recall and copy data from the Rey figure as a measure of central coherence [32]. Contrary to other findings, this study did not however, find any differences between recovered AN and the HC group [33]. The difference in effect size between currently ill ANs and recovered can be observed in Fig 2, with recovered ANs demonstrating a more global processing style than the AN group. Although this data lends support to the notion that such characteristics may be influenced by starvation, we are limited in making further interpretations due to the cross-sectional design of this study. Studies employing longitudinal designs are needed to examine this thought further.

One rather curious finding from this study are the rather contradicting findings of poorer global processing in unaffected relatives of those with AN and better global processing in recovered ANs in comparison to currently ill ANs. One of these findings lends support to such characteristics representing an underlying vulnerability, whereas the other suggests they are a consequence of starvation. One possible explanation for high levels of global processing in the recovered AN group in this study is that it is likely that a large proportion of these individuals received Cognitive Remediation Therapy (CRT) as part of their treatment package. Involvement in such cognitive training in combination with weight-gain may therefore have significantly impacted on this groups processing style, either through increasing insight into alternative strategies or by learning to employ alternative ways of processing information. Such explanations of the current findings are speculative; however they are supported by the findings of RCTs demonstrating cognitive improvement following CRT in adults with AN [34].

Furthermore, this study did not find any significant correlations between clinical variables and central coherence index. Such findings may indicate that poor global processing is independent of such factors and maybe an underlying trait.

The findings from this study are important for the field of ED for several reasons. Firstly, as the Rey Figure is one of the most widely used neuropsychological measures of CC in ED, this study provides much needed normative data that can be used as a benchmark for clinicians and future studies. These data address the methodological flaws in the current literature, which have led to inconsistent results and discrepant findings between studies. Furthermore, employing a consistent administration and scoring system and reporting the data in a systematic fashion helps to synthesise knowledge, identify consistencies and provides robust comparison data for future studies. This is an important issue in clinical trials where neuropsychological assessments are used as an outcome measure and it may not be possible to recruit a healthy control group. The reliable data presented in the form of means standard deviations and medians reported in this manuscript can therefore be used for comparisons. Secondly, the different processing profiles identified across the ED sub-types provide strong support for the Rey Figure as a sensitive measure of central coherence within ED. The use of a scoring system to derive the central coherence index based on measures of order and style produces a reliable outcome. We suggest that in order to avoid inconsistencies, the use and development of alternative measures and scoring systems should be discouraged.

One factor that may contribute to the differences in outcomes produced between studies using Booth’s scoring system is the scoring of the style index. This index relies a little more on experimenter’s judgment and therefore may be vulnerable to error or bias. It is therefore recommended that researchers are fully trained in the administration and scoring of the Rey Figure and clear guidelines are given as to what constitutes certain scores. It is also recommended that as well as video recording the administration of Rey Figures, future research studies should also attempt to co-rate a minimum of 10% of their data and perform reliability checks to be reported in published manuscripts.

This paper also provides the largest dataset for the Rey Figure with both individuals with BN and also unaffected family members of those with AN. The findings demonstrated poorer global processing in these groups compared to HC comparisons, and a similar global processing style to what is observed in AN. Such findings provide support for the development and use of remediation treatments with both clinical groups. Cognitive Remediation Therapy (CRT) directly targets biased cognitive processing, such as poor global processing, and randomised controlled trials have shown improvements to cognitive processing following CRT in individuals with AN [35]. However, due to the limited amount of research in BN, CRT has not been routinely implemented with this group. The results of the present study provide strong evidence to suggest that CRT could also be of potential benefit for BN, as suggested by Dingemans et al., (2013) [36].

Furthermore, unhelpful cognitive styles within families, such as lower levels of global processing highlighted here are likely to negatively impact on the responses of carers to ED and may produce maladaptive behaviours, such as a rigid parenting style and an inability to see the wider context of the problem, that may inadvertently serve to maintain the ED. These findings indicate that including family members in treatments such as CRT may be beneficial for helping to raise awareness of cognitive processing styles within families, and could result in more adaptive coping to ED pathology. In support of this a small pilot study demonstrated that a module of CRT developed for collaborative use with individuals with AN and their carers was feasible [37,38].

The strengths of this study include its large sample size and inclusion of ED subtypes and unaffected relatives. Additionally, the collated data presented here holds advantages over that of a meta-analysis as the raw data from each study was collated and analysed, compared to the use of sample averages in a meta-analysis. This analysis allowed for the production of means and medians that can be used as benchmark data for future studies. Collating the data in this fashion has been done previously in AN with regards to set-shifting, and have proved to be extremely useful for the ED field [1,39].

As the results for this study were obtained by combining data from several studies one potential weakness is the lack of a consistent measure of ED pathology or intelligence. The studies from which the data were collated were investigating different hypotheses and employed different measures to do so. It was therefore not possible to report on these characteristics in the present study. In terms of limitations, it is also acknowledged that this paper reports on a re-analysis of previously published datasets. The fact that this study only considers data from one research group could also be seen as a potential weakness, for example as mentioned above a number of patients within the studies are likely to have been offered CRT as part of their treatment package, which may have contributed to the outcomes in the recovered AN group and may therefore not necessarily generalise to other recovered AN populations. However, this is also a strength of the study as we can be sure that the Rey was administered and scored consistently, adding validity and reliability to this studies results.

In conclusion, this study provides robust data from a large sample size for perhaps the most widely used measure of central coherence in the ED literature. Reporting data in this synthesised fashion provides reliable normative data useful for future research studies within ED and the wider psychiatric field.
